# Logistic regression analysis on risk factors of augmented vertebra recompression after percutaneous vertebral augmentation

**DOI:** 10.1186/s13018-021-02480-9

**Published:** 2021-06-11

**Authors:** Zhongcheng An, Chen Chen, Junjie Wang, Yuchen Zhu, Liqiang Dong, Hao Wei, Lianguo Wu

**Affiliations:** 1 Department of Spinal Surgery, The Second Affiliated Hospital of Zhejiang Chinese Medical University, Hangzhou, 310005 Zhejiang People’s Republic of China; 2 The Second Clinical Medical College of Zhejiang Chinese Medical University, Hangzhou, 310005 Zhejiang People’s Republic of China

**Keywords:** Osteoporotic vertebral compression fracture, Percutaneous vertebral augmentation, Augmented vertebra recompression, Risk factors, Logistic regression analysis

## Abstract

**Objective:**

To explore the high-risk factors of augmented vertebra recompression after percutaneous vertebral augmentation (PVA) in the treatment of osteoporotic vertebral compression fracture (OVCF) and analyze the correlation between these factors and augmented vertebra recompression after PVA.

**Methods:**

A retrospective analysis was conducted on 353 patients who received PVA for a single-segment osteoporotic vertebral compression fracture from January 2017 to December 2018 in our department according to the inclusion criteria. All cases meeting the inclusion and exclusion criteria were divided into two groups: 82 patients in the recompression group and 175 patients in the non-compression group. The following covariates were reviewed: age, gender, body mass index (BMI), injured vertebral segment, bone mineral density (BMD) during follow-up, intravertebral cleft (IVC) before operation, selection of surgical methods, unilateral or bilateral puncture, volume of bone cement injected, postoperative leakage of bone cement, distribution of bone cement, contact between the bone cement and the upper or lower endplates, and anterior height of injured vertebrae before operation, after surgery, and at the last follow-up. Univariate analysis was performed on these factors, and the statistically significant factors were substituted into the logistic regression model to analyze their correlation with the augmented vertebra recompression after PVA.

**Results:**

A total of 257 patients from 353 patients were included in this study. The follow-up time was 12–24 months, with an average of 13.5 ± 0.9 months. All the operations were successfully completed, and the pain of patients was relieved obviously after PVA. Univariate analysis showed that in the early stage after PVA, the augmented vertebra recompression was correlated with BMD, surgical methods, volume of bone cement injected, preoperative IVC, contact between bone cement and the upper or lower endplates, and recovery of anterior column height. The difference was statistically significant (*P* < 0.05). Among them, multiple factors logistic regression elucidated that more injected cement (*P* < 0.001, OR = 0.558) and high BMD (P = 0.028, OR = 0.583) were negatively correlated with the augmented vertebra recompression after PVA, which meant protective factors (B < 0). Preoperative IVC (*P* < 0.001, OR = 3.252) and bone cement not in contact with upper or lower endplates (*P* = 0.006, OR = 2.504) were risk factors for the augmented vertebra recompression after PVA. The augmented vertebra recompression after PVP was significantly less than that of PKP (*P* = 0.007, OR = 0.337).

**Conclusions:**

The augmented vertebra recompression after PVA is due to the interaction of various factors, such as surgical methods, volume of bone cement injected, osteoporosis, preoperative IVC, and whether the bone cement is in contact with the upper or lower endplates.

With the progress of population aging and the numbers of old people increasing, the incidence rate of osteoporotic vertebral compression fracture (OVCF) rises year by year, which has a serious impact on the life of patients, and receives increasing attention [1, 2]. Percutaneous vertebral augmentation (PVA) represented by percutaneous vertebroplasty (PVP) and percutaneous kyphoplasty (PKP), which has high safety and reliable short-term efficacy, has become the most commonly used treatment for OVCF in recent years [3]. However, with the widespread application of PVA, some patients have lost the height of the injured vertebra again after surgery (without trauma), resulting in the vertebral height returning to the preoperative status, and even kyphosis deformity, which requires revision surgery when it is serious [4]. Therefore, it has become increasingly important to correctly understand the reasons for the augmented vertebra recompression after PVA.

Some scholars found that the augmented vertebra recompression after PVA might be related to osteoporosis, osteonecrosis, intravertebral cleft (IVC) before PVA, bone cement distribution, excessive correction of anterior column height, insufficient correction of kyphosis, etc. [5, 6]. But the specific reasons are still unclear. Therefore, it is of great clinical significance to determine the exact reasons for the augmented vertebra recompression after PVP to ensure the long-term efficacy of patients. Our study retrospectively analyzed the clinical data of 353 patients with OVCF who underwent PVA in our hospital from January 2017 to December 2018 and used logistic regression analysis to analyze the main risk factors of the augmented vertebra recompression after PVA in order to provide effective prevention and treatment measures to prevent postoperative vertebral collapse and improve the surgical efficacy.

## Materials and methods

### Inclusion and exclusion criteria

The following are the inclusion criteria: (1) meeting the diagnostic criteria of osteoporosis [7], (2) OVCF caused by acute or subacute non-significant external force, (3) only one vertebral fracture, (4) follow-up for more than 12 months with complete follow-up data, (5) no obvious trauma history during follow-up, (6) underwent PVA treatments, and (7) no complications related to PVA, such as pulmonary embolism.

The following are the exclusion criteria: (1) pathological vertebral fracture caused by tumor or infectious disease, (2) neurological impairment before and after PVA, (3) intraspinal space-occupying caused by burst fracture, (4) history of spinal internal fixation surgery, (5) history of bone metabolic diseases except osteoporosis, and (6) treated with conservative therapy.

### General information

**Fig. 1:**
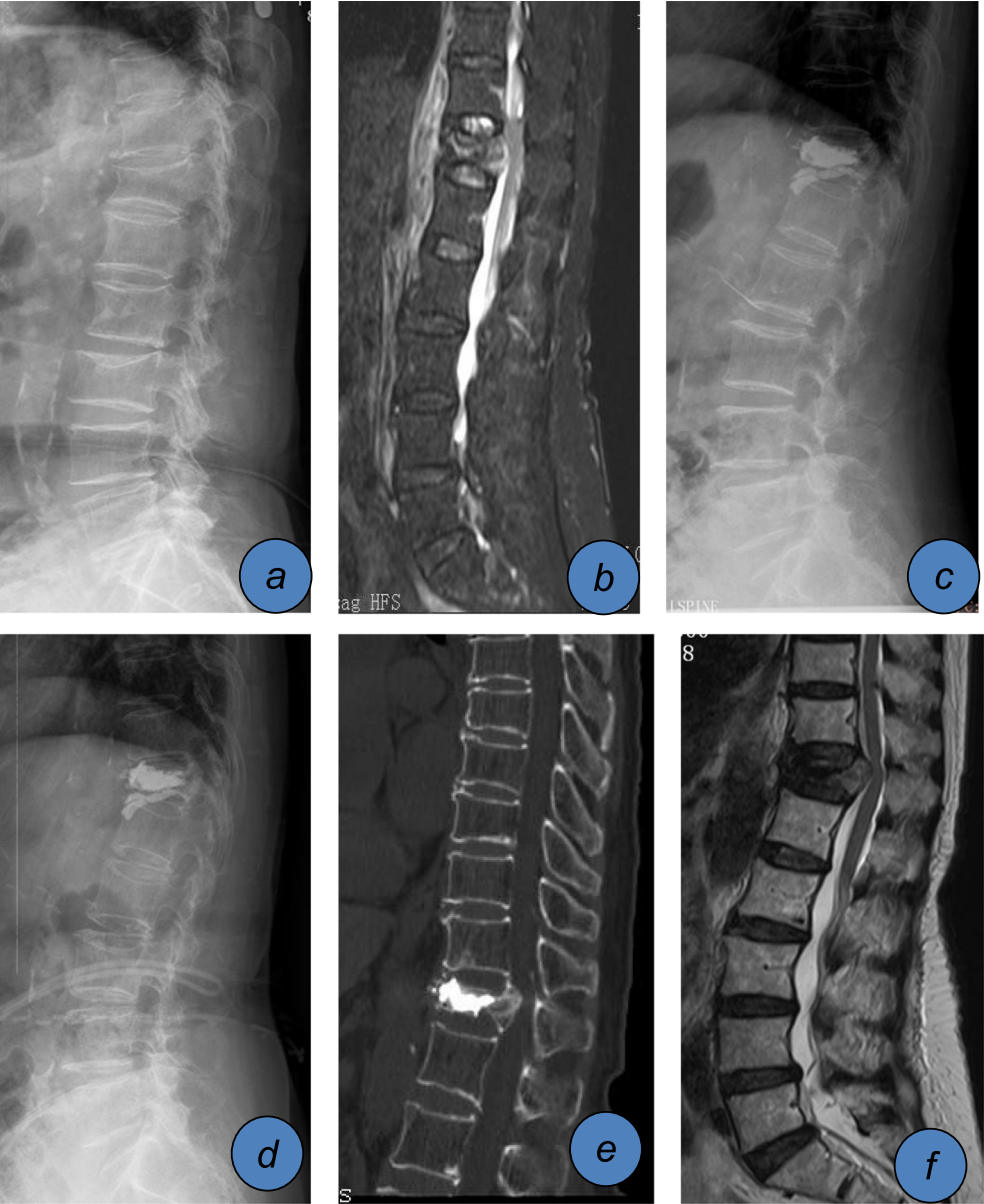
An 80-year-old female patient with thoracic 12 vertebral compression fractures underwent PKP. BMD T = − 3.3. **a** X-ray showed a wedge-shaped change of T12. **b** MR showed a fresh compression fracture of T12, with IVC. **c** After PKP surgery, the X-ray showed that there was no obvious collapse of the T12 vertebral body. **d** After 3 months, the X-ray showed that the T12 vertebral body had local collapse and kyphosis aggravation. **e** The sagittal plane CT showed that the T12 vertebral body collapsed and the posterior wall of the vertebral body squeezed the spinal canal. **f** MR showed that the spinal canal and spinal cord were compressed

A total of 353 patients with single-segment OVCF who underwent PVA from January 2017 to December 2018 in our department were collected. The fracture sites were divided into the thoracic region (T5-T10), thoracolumbar region (T11-L2), and lumbar region (below L3). A total of 257 patients who met the above criteria were included in this study. There were 67 men and 190 women aged from 55 to 90 years old (mean 74.96 ± 11.48). All patients were examined by X-ray, computed tomography (CT), and magnetic resonance imaging (MR) before PVA (Fig. 1). All investigations were approved by the hospital ethics committee (ethical approval number: 2020-kl-040-01).

### Grouping and clinical study

Patients who met the inclusion and exclusion criteria were retrospectively studied. The standard of the augmented vertebra recompression after PVA was determined by the loss of injured vertebra height ≥ 15% or the increase of vertebral local kyphosis angle ≥10° at the last follow-up [6]. According to the above criteria, the patients were divided into two groups: 82 patients in the recompression group and 175 patients in the non-compression group. We collected age, gender, BMI, injured vertebral segment, BMD during follow-up, preoperative IVC, selection of surgical methods, unilateral or bilateral puncture, volume of bone cement injected, leakage of bone cement, distribution of bone cement, contact between the bone cement and the upper or lower endplates, and anterior height of injured vertebrae before operation, after surgery, and at the last follow-up.

### Statistical methods

The SPSS 23.0 software was used for statistical analysis of the data. Measurement data were expressed by x̅ ± s, group comparison using t test, and counting material using chi-square test, and *P* < 0.05 was considered statistically significant. Finally, all the observation indexes were taken as single factor covariates, and whether there was the augmented vertebra recompression after PVA was taken as the dependent variable, and the logistic regression model was used for multivariate analysis. The 95% confidence interval (CI), *P* < 0.05was considered to be statistically significant.

## Results

A total of 257 patients who met the inclusion and exclusion criteria were included in this study. All included patients selected surgical indications for treatment according to the assessment system for evaluating the severity of thoracolumbar osteoporotic fracture [8], and all operations went smoothly, without complications such as infection, bleeding, neural functional injury, vascular embolism, and bone cement reaction.

### Univariate analysis of the factors related to the augmented vertebra recompression after PVA

In the early stage after PVA, the augmented vertebra recompression after PVA was correlated with BMD, surgical methods, volume of bone cement injected, whether there were IVC before PVA, whether the bone cement is in contact with the upper or lower endplates, and recovery of anterior column height, and the difference was statistically significant (*P* < 0.05). The specific analysis results are shown in Table 1.

**Table 1:** Univariate analysis of the factors related to the augmented vertebra recompression after PVA

	Recompression group	Non-compression group	T/x^2^ value	*P* value
**Age**	74.78 ± 11.76	75.05 ± 11.38	−0.176	0.860
**Gender**			0.245	0.621
Male	23	44		
Female	59	131		
**BMI**	22.13 ± 4.94	22.35 ± 6.03	−0.290	0.772
**BMD**	−2.46 ± 0.68	−1.88 ± 0.72	−6.091	0.000*
**Vertebral distribution**			2.589	0.274
Thoracic region	22	32		
Thoracolumbar region	50	116		
Lumbar region	10	27		
**IVC**			25.826	0.000*
Yes	49	47		
No	33	128		
**Surgical methods**			18.499	0.000*
PKP	70	102		
PVP	12	73		
**Unilateral or bilateral puncture**			2.620	0.106
Unilateral	44	75		
Bilateral	38	100		
**Leakage of bone cement**			0.018	0.892
Yes	34	71		
No	48	104		
**The bone cement was in contact with the upper or lower endplates**			17.731	0.000*
Yes	53	64		
No	29	111		
**Volume of bone cement injected (ml)**	3.34 ± 1.26	4.55 ± 1.41	−6.951	0.000*
**Distribution of bone cement**			1.725	0.189
Type H	38	66		
Type O	44	109		
**Recovery of anterior column height (%)**	13.62 ± 8.76	16.42 ± 11.18	−2.002	0.046*

### Logistic regression analysis of the factors related to the augmented vertebra recompression after PVA

Based on the result of univariate factor analysis, variables which were statistically different were put into following logistic regression analysis. Among them, more injected cement (*P* < 0.001, OR = 0.558) and high BMD (*P* = 0.028, OR = 0.583) were negatively correlated with the augmented vertebra recompression after PVA, which were the protective factors (B < 0). IVC before PVA (*P* < 0.001, OR = 3.252) and bone cement not in contact with upper or lower endplates (*P* = 0.006, OR = 2.504) were risk factors for the augmented vertebra recompression after PVA. The incidence of the augmented vertebra recompression after PVP was significantly less than that of PKP (*P* = 0.007, OR = 0.337). The specific analysis results are shown in Table 2.

**Table 2:** Logistic regression analysis of the factors related to the augmented vertebra recompression after PVA

	B value	Se value	Wald value	P value	Or value	95%CI
BMD	−0.539	0.245	4.853	0.028*	0.583	0.361~0.942
IVC	1.179	0.331	12.724	0.000*	3.252	1.701~6.217
Surgical methods	− 1.089	0.407	7.166	0.007*	0.337	0.152~0.747
The bone cement was in contact with the upper or lower endplates	0.918	0.335	7.520	0.006*	2.504	1.299~4.827
Volume of bone cement injected (ml)	− 0.584	0.142	16.873	0.000*	0.558	0.422~0.737
Recovery of anterior column height (%)	− 0.008	0.016	0.253	0.615	0.992	0.961~1.024

## Discussion

PVA has been widely applied to treat OVCF because of its rapid and exact effect on pain relief and functional improvement. However, the augmented vertebra recompression is a common postoperative complication of PVA, which has attracted more and more attention from clinicians. Scholars have not unified judgment criteria for augmented vertebra recompression after PVA; therefore, the reported incidence has great difference [6, 9, 10]. At present, in clinical studies, the vertebral height loss ≥15% or local kyphosis angle increase ≥10° are mostly used as the diagnostic criteria for augmented vertebra recompression after PVA. In our study, a total of 257 patients were included, 82 patients suffered from augmented vertebra recompression after PVA, with an incidence of about 31.91%.

### Relationship between the augmented vertebra recompression after PVA and surgical methods (PVP/PKP)

A large number of studies have shown that PVP and PKP have similar clinical efficacy in the treatment of OVCF and do not increase the incidence of adjacent vertebral re-fracture [11, 12]. However, some scholars have shown that PKP was an important risk factor for the augmented vertebra recompression after PVA. Kim et al. [13] used osteoporotic cadaveric fractured vertebral bodies to conduct an ex vivo biomechanical study and confirmed that the average height loss of PKP was significantly greater than that of PVP (4.2 mm vs 1.1 mm). Li et al. [14] reviewed the patients who underwent PKP or PVP and found that the postoperative vertebral height of the PKP group was higher than that of the PVP group. This is similar to the results of our study. The reason may be that in the PVP group, the bone cement can diffuse more easily to the non-fracture area and endplate through the trabecular space, occlude the surrounding cancellous bone more tightly, and the bone cement diffusion range is wider, so that the vertebrae can obtain better anti-compression ability. However, when PKP is selected, the cancellous bone in the injured vertebral body will be compressed to form a bone barrier during balloon dilatation, which affects the uniform diffusion of bone cement around the injured vertebral body, causing the bone cement injected into the body to form a lump and combine with surrounding tissues loosely. Besides, it will increase the stress of the upper and lower edge of the vertebral body, and greatly increases the risk of loss of injured vertebral height after the operation.

### Relationship between the augmented vertebra recompression after PVA and bone cement (injection amount, distribution, and contact with upper and lower endplates)

The amount of bone cement injected and the distribution of bone cement in the injured vertebrae have always been the focus of attention. Recently, some scholars found that lump distribution of bone cement mass is an important risk factor for the augmented vertebra recompression after PVA [5, 15, 16]. Kim et al. [13] confirmed by an ex vivo biomechanical study that the stiffness of the vertebral body with lump cement distribution is significantly lower than that of the bone cement through the bone trabecular space distribution, which makes the cancellous bone around the lump bone cement easier to recompress. Some scholars also found that the presence of non-PMMA-endplate-contact (NPEC) plays an important role in inducing the augmented vertebra recompression after PVA. Li et al. [17] considered that the distance between the bone cement and the upper or lower endplates of the injured vertebrae can reflect the dispersion degree of bone cement more accurately. If the distance is too large, the risk of the augmented vertebra recompression will be increased, and the augmented vertebra recompression mainly occurs in the upper part of the vertebral body where it is not filled with bone cement. Therefore, more and more scholars emphasize that the bone cement should be distributed evenly as far as possible, and the upper and lower endplates should be contacted by bone cement together to avoid recompression of the augmented vertebra. Contact between the bone cement and the upper and lower endplates together is an important independent protective factor for recompression of the augmented vertebrae after PVA. This is similar to the results of a recent meta-analysis [18]. Our results also found that within a certain range, the more bone cement injected, the lower incidence of the augmented vertebra recompression occurred after PVA. Furthermore, we also found the incidence of augmented vertebra recompression in patients with NPEC was significantly higher than that in patients without NPEC in our study. Therefore, we suggest that bone cement should contact the upper and lower endplates in the injured vertebrae as much as possible, and achieve a “foot on the ground, head on the dome” visual effect on the X-ray to strengthen the anchoring effect of bone cement and trabecula of injured vertebrae.

PVA can be injected into the bone cement through unilateral or bilateral approaches. The current meta-analyses showed that the clinical efficacy of the unilateral approach is similar to that of the bilateral approach [19]. However, the bilateral approach can inject a large amount of bone cement and has better biomechanical stability. Vitro biomechanical study showed that the bilateral approach could make the bone cement present an “H” shape distribution on both sides of the vertebral body and generated steady supporting force, and established an effective biomechanical balance, which was more in line with the normal mechanical conduction state of the human body [20]. Previously, some scholars believed that the biased distribution of bone cement to one side affected the stress distribution in the vertebral body. They believed that the unilateral pedicle of vertebral arch approach injection of bone cement mostly showed an “O” shape distribution, and it was difficult to achieve a symmetrical bone cement distribution on both sides of the vertebral body. They believed that unilateral injection of bone cement often presents an “O” shape, which makes it difficult to achieve symmetrical distribution on both sides of the vertebral body, resulting in unilateral load-bearing of the vertebral body and biomechanical imbalance of the bilateral vertebral body. Under a constant load, the side with weaker biomechanics was prone to collapse again [21, 22]. However, clinical studies have shown that unilateral bone cement filling reaching the midline is enough. So, most scholars accept unilateral injection, but it is required to inject bone cement beyond or as closer to the midline as possible [23–25].

### Relationship between the augmented vertebra recompression after PVA and osteoporosis

Previous studies have shown that decreased BMD is the main cause of vertebral re-fracture after PVA [26, 27]. In addition, Li et al. [14] found that low BMD was closely related to the augmented vertebra recompression after PVA. The vertebral trabeculae are sparse in patients with osteoporosis, the strength and anti-compressive of the vertebral body are significantly reduced, and then complications such as height reduction and kyphosis usually occur. Besides, there is acute bone loss at fracture site after trauma, while anti-osteoporosis drug can not improve BMD in a short time. Even if there is no obvious trauma, the augmented vertebra recompression in daily activities after PVA is inevitable. The results of our study showed that BMD was an important risk factor for the augmented vertebra recompression after PVA. Logistic regression also confirmed that the decrease of BMD could significantly increase the loss of the augmented vertebra recompression after PVA. Therefore, regular anti-osteoporosis treatment is of great significance to reduce the incidence of the augmented vertebra recompression after PVA.

### Relationship between the augmented vertebra recompression after PVA and osteonecrosis and IVC

Some previous research has suggested that temperature elevations during cement curing may induce irreversible damage of the bone and surrounding tissue, or bone cement may diffuse into and embolize the blood vessels in the vertebral body, resulting in avascular necrosis of the vertebral body in the corresponding area and leading to the collapse of the vertebral body or re-fracture [28, 29]. Heo et al. [30] made a study on the recompression of augmented vertebrae after PVP and found that the incidence of the recompression of augmented vertebrae with osteonecrosis (28.57%) was significantly higher than that of the surgical vertebral body without osteonecrosis (1.24%). Therefore, it is considered that osteonecrosis in the surgical vertebral body is an important risk factor for the augmented vertebrae recompression after PVA.

In recent years, many scholars believe that the existence of IVC may be an important risk factor for the augmented vertebrae recompression after PVA, and IVC is also considered to be a characteristic manifestation of osteonecrosis in imaging [4, 6]. According to a recent meta-analysis, IVC was an important reason for the recompression of the vertebrae after PVA [18]. Meanwhile, Yu et al. [31] also pointed out that the existence of IVC was crucial to predicting postoperative vertebral body recompression. Through our study, we found that the recompression of the augmented vertebral after PVA is more common in patients with IVC before operation. Therefore, we have reason to believe that the existence of IVC has an important impact on the augmented vertebrae recompression after PVA, and patients with preoperative IVC have a significantly higher risk of recompression of injured vertebra after PVA than patients without IVC. At the same time, we also have every reason to believe that IVC and osteonecrosis may have similar mechanisms in the occurrence of the augmented vertebrae recompression after PVA.

## Limitations

This study has the following limitations: First, this study is a retrospective study. Second, the surgeons are not the same doctor, so the operation process can not be completely consistent. Finally, some scholars found that most of the re loss of injured vertebral height mainly occurred within 3 months after operation [9], so the follow-up time of this study was selected as 12–24 months, but a longer follow-up time is needed to ensure the risk factors of augmented vertebra recompression after percutaneous more comprehensively. At the same time, it should be noted that due to the diversity of patients, medical conditions, surgical methods, research methods and follow-up time, and other variable factors, new potential risk factors have been put forward continuously, so the reliability of the above conclusions still needs further prospective studies to be confirmed.

## Conclusions

To sum up, the augmented vertebrae recompression after PVA is due to the interaction of various factors, such as surgical methods, volume of bone cement injected, NPEC, osteoporosis, and IVC, which provides a certain reference for the prevention of postoperative vertebral recompression.

## Data Availability

The datasets generated and analyzed during the current study are available from the corresponding author on reasonable request.

## Abbreviations

PVA: Percutaneous vertebral augmentation
OVCF: Osteoporotic vertebral compression fracture
BMI: Body mass index
BMD: Bone mineral density
IVC: Intravertebral cleft
PVP: Percutaneous vertebroplasty
PKP: Percutaneous kyphoplasty
NPEC: Non-PMMA-endplate-contact
CT: Computed tomography
MR: Magnetic resonance imaging
